# *catena*-Poly[[(1,10-phenanthroline)cobalt(II)]-di-μ-azido]

**DOI:** 10.1107/S1600536812006435

**Published:** 2012-02-24

**Authors:** Xue-Miao Gao, Jiong-Peng Zhao, Fu-Chen Liu

**Affiliations:** a School of Chemistry and Chemical Engineering, Tianjin University of Technology, Tianjin 300384, People’s Republic of China

## Abstract

In the crystal structure of the binuclear title complex, [Co(N_3_)_2_(C_12_H_8_N_2_)]_*n*_, each Co^II^ cation is coordinated by two N atoms from one chelating 1,10-phenanthroline ligand and four azide ligands in a slightly distorted octa­hedral coordination. The two Co^II^ cations of the binuclear complex are related by an inversion centre and are bridged by two symmetry-related azide ligands in both μ_1,1_ and μ_1,3_ modes. The μ_1,3_ bridging mode gives rise to an infinite one-dimensional chain along the *a* axis, whereas the μ_1,1_ bridging mode is responsible for the formation of the binuclear Co^II^ complex.

## Related literature

For general background to metal–azide complexes, see: Zhao *et al.* (2009 ▶). For a closely related Ni–azide structure, see: Li *et al.* (2000 ▶). 
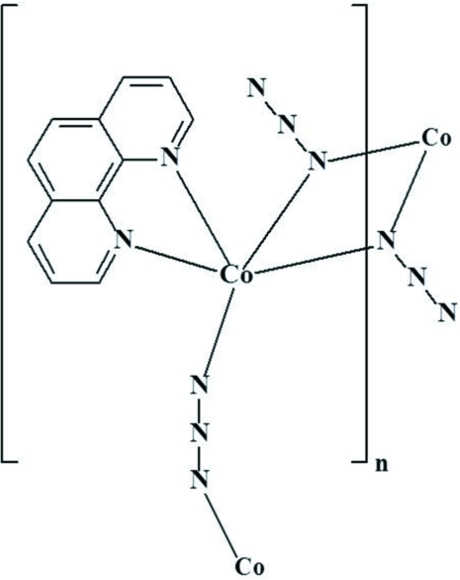


## Experimental

### 

#### Crystal data

[Co(N_3_)_2_(C_12_H_8_N_2_)]
*M* *_r_* = 323.19
Triclinic, 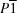

*a* = 7.0018 (14) Å
*b* = 10.049 (2) Å
*c* = 10.491 (2) Å
α = 109.83 (3)°
β = 103.63 (3)°
γ = 105.78 (3)°
*V* = 622.8 (2) Å^3^
*Z* = 2
Mo *K*α radiation
μ = 1.38 mm^−1^
*T* = 293 K
0.2 × 0.18 × 0.18 mm

#### Data collection

Rigaku SCXmini diffractometer
Absorption correction: multi-scan (*ABSCOR*; Higashi, 1995 ▶) *T*_min_ = 0.720, *T*_max_ = 1
6534 measured reflections
2822 independent reflections
2087 reflections with *I* > 2σ(*I*)
*R*_int_ = 0.053
Standard reflections: 0

#### Refinement

*R*[*F* ^2^ > 2σ(*F* ^2^)] = 0.052
*wR*(*F* ^2^) = 0.101
*S* = 1.07
2822 reflections
190 parameters
H-atom parameters constrained
Δρ_max_ = 0.36 e Å^−3^
Δρ_min_ = −0.42 e Å^−3^

### 

Data collection: *PROCESS-AUTO* (Rigaku, 1998 ▶); cell refinement: *PROCESS-AUTO*; data reduction: *PROCESS-AUTO*; program(s) used to solve structure: *SHELXS97* (Sheldrick, 2008 ▶); program(s) used to refine structure: *SHELXL97* (Sheldrick, 2008 ▶); molecular graphics: *ORTEPIII* (Burnett & Johnson, 1996 ▶) and *PLATON* (Spek, 2009 ▶); software used to prepare material for publication: *SHELXTL* (Sheldrick, 2008 ▶).

## Supplementary Material

Crystal structure: contains datablock(s) global, I. DOI: 10.1107/S1600536812006435/vn2030sup1.cif

Structure factors: contains datablock(s) I. DOI: 10.1107/S1600536812006435/vn2030Isup2.hkl

Additional supplementary materials:  crystallographic information; 3D view; checkCIF report

## Figures and Tables

**Table 1:** Selected bond lengths (Å)

Co1—N1^i^	2.113 (3)
Co1—N1	2.175 (3)
Co1—N4	2.202 (3)
Co1—N6^ii^	2.144 (3)
Co1—N7	2.141 (3)
Co1—N8	2.141 (3)

Symmetry codes: (i) 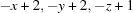; (ii) 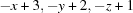.

**Fig. 1.**
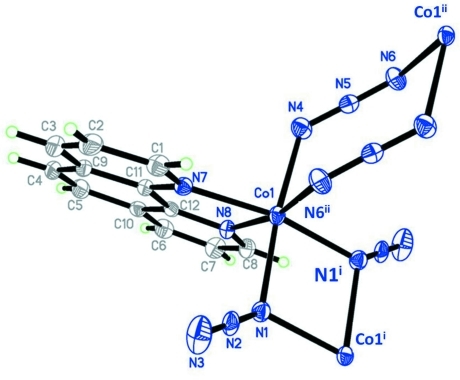
The coordinated mode and linkage of the complex. Atomic displacement ellipsoids are drawn at the 30% probability level. Symmetry codes: (i) 2 - x, 2 - y, 1 - z; (ii) 3 - x, 2 - y, 1 - z.

**Fig. 2.**
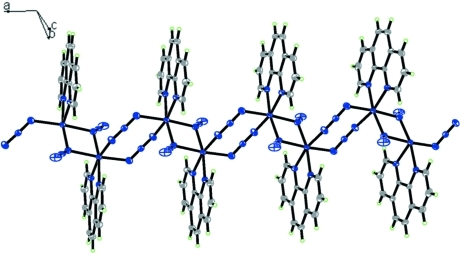
The 1D chain of the complex along the a-axis.

**Fig. 3.**
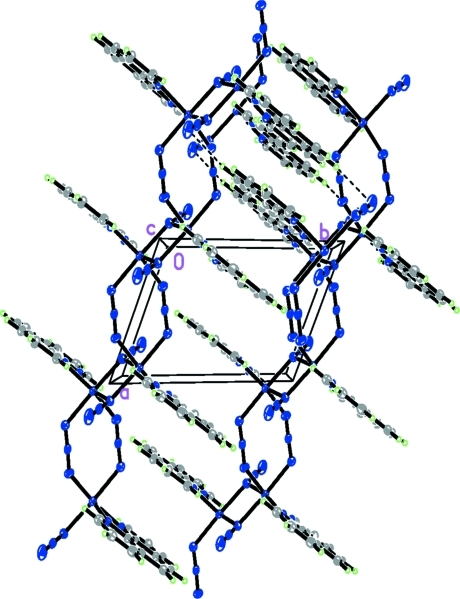
Packing plot of the title compound. The dotted lines indicate the weak hydrogen bonds between the azide anions and ring H atoms of adjacent chains

## supplementary crystallographic information

### Comment

Some metal-azido complexes with unique structural features have been reported in the past (Zhao *et al.*, 2009). Co-ligands are often encountered in metal azido complex, *e.g.* the 1,10-phenanthroline ligand which are used frequently in assembling metal azido complexes. One 1D nickel-azido complex with 1,10-phenanthroline as co-ligand was reported in 2000 (Li *et al.*, 2000). However, its isomorphic Co^II^ compound was not reported until this work. That may be due to the fact that Co^II^ cations are easy oxidated to Co^III^ with 1,10-phenanthroline as co-ligand. In the title complex the Co^II^ ion is coordinated by one chelating 1,10-phenanthroline and four azido anions forming a distorted CoN_6_ octahedral environment (Fig. 1). The azido anions take two different coordinated types, in which one bridges two Co^II^ ions in µ_1,1_ mode while the other one bridges two Co^II^ ions in µ_1,3_ mode. The two µ_1,1_ azido anions link two Co^II^ ions forming a binuclear dimer whereas the dimers linked by the two µ_1,3_ azido anions yield a 1D chain of bunuclear Co^II^ complexes (Fig. 2). π–π stacking of the phenanthroline aromatic rings between adjacent chains assists in the forming of a 3D supermolecular structure (Fig. 3). The smallest centroid to centroid distances between the aromatic rings of phenanthroline aromatic rings between adjacent chains are 3.589 (2) Å and 3.605 (2) Å, respectively. A weak CH···N interaction is present between the aromatic H5 proton and the terminal N3 azide nitrogen (2.54 Å), reinforcing slightly the packing of adjacent chains.

### Experimental

The complex was hydrothermally synthesized under auto-generated pressure. A mixture of cobalt formate (1 mmol), NaN_3_ (1 mmol) and 1,10-phenanthroline (1 mmol) in methanol was sealed in a Teflon-lined stainless-steel Parr bomb that was heated at 413 K for 48 h. Red crystals of the title complex were collected after the bomb was allowed to cool to room temperature. Yield 20% based on metal salt.

### Refinement

H atoms were included in calculated positions and treated as riding on their parent C atoms with C—H = 0.93Å and *U*_iso_(H) = 1.2*U*_eq_(C).

### Crystal data

**Table:**

[Co(N_3_)_2_(C_12_H_8_N_2_)]	*Z* = 2
*M**_r_* = 323.19	*F*(000) = 326
Triclinic, *P*1	*D*_x_ = 1.723 Mg m^−^^3^
Hall symbol: -P 1	Mo *K*α radiation, λ = 0.71073 Å
*a* = 7.0018 (14) Å	Cell parameters from 5775 reflections
*b* = 10.049 (2) Å	θ = 3.2–27.5°
*c* = 10.491 (2) Å	µ = 1.38 mm^−^^1^
α = 109.83 (3)°	*T* = 293 K
β = 103.63 (3)°	Block, red
γ = 105.78 (3)°	0.2 × 0.18 × 0.18 mm
*V* = 622.8 (2) Å^3^	

### Data collection

**Table:**

Rigaku SCXmini diffractometer	2822 independent reflections
Radiation source: fine-focus sealed tube	2087 reflections with *I* > 2σ(*I*)
Graphite monochromator	*R*_int_ = 0.053
ω scans	θ_max_ = 27.5°, θ_min_ = 3.2°
Absorption correction: multi-scan (*ABSCOR*; Higashi, 1995)	*h* = −9→9
*T*_min_ = 0.720, *T*_max_ = 1	*k* = −13→13
6534 measured reflections	*l* = −13→13

### Refinement

**Table:**

Refinement on *F*^2^	Primary atom site location: structure-invariant direct methods
Least-squares matrix: full	Secondary atom site location: difference Fourier map
*R*[*F*^2^ > 2σ(*F*^2^)] = 0.052	Hydrogen site location: inferred from neighbouring sites
*wR*(*F*^2^) = 0.101	H-atom parameters constrained
*S* = 1.07	*w* = 1/[σ^2^(*F*_o_^2^) + (0.0411*P*)^2^ + 0.0273*P*] where *P* = (*F*_o_^2^ + 2*F*_c_^2^)/3
2822 reflections	(Δ/σ)_max_ = 0.001
190 parameters	Δρ_max_ = 0.36 e Å^−^^3^
0 restraints	Δρ_min_ = −0.42 e Å^−^^3^

### Special details

**Table:**

Geometry. All esds (except the esd in the dihedral angle between two l.s. planes) are estimated using the full covariance matrix. The cell esds are taken into account individually in the estimation of esds in distances, angles and torsion angles; correlations between esds in cell parameters are only used when they are defined by crystal symmetry. An approximate (isotropic) treatment of cell esds is used for estimating esds involving l.s. planes.
Refinement. Refinement of F^2^ against ALL reflections. The weighted R-factor wR and goodness of fit S are based on F^2^, conventional R-factors R are based on F, with F set to zero for negative F^2^. The threshold expression of F^2^ > 2sigma(F^2^) is used only for calculating R-factors(gt) etc. and is not relevant to the choice of reflections for refinement. R-factors based on F^2^ are statistically about twice as large as those based on F, and R- factors based on ALL data will be even larger.

### Fractional atomic coordinates and isotropic or equivalent isotropic displacement parameters (Å^2^)

**Table:**

	*x*	*y*	*z*	*U*_iso_*/*U*_eq_	
Co1	1.08955 (7)	0.88775 (5)	0.39668 (4)	0.02424 (16)	
N1	0.8499 (4)	0.9870 (3)	0.3808 (3)	0.0290 (6)	
N2	0.7988 (4)	1.0243 (3)	0.2840 (3)	0.0321 (7)	
N3	0.7510 (6)	1.0590 (4)	0.1905 (4)	0.0599 (11)	
N4	1.3416 (5)	0.7993 (3)	0.4387 (3)	0.0357 (7)	
N5	1.5146 (5)	0.8649 (3)	0.5268 (3)	0.0264 (6)	
N6	1.6882 (5)	0.9262 (3)	0.6149 (3)	0.0362 (7)	
N7	0.9662 (4)	0.7562 (3)	0.1657 (3)	0.0249 (6)	
N8	0.8808 (4)	0.6720 (3)	0.3697 (3)	0.0249 (6)	
C1	1.0005 (6)	0.8017 (4)	0.0652 (4)	0.0338 (8)	
H1A	1.0859	0.9035	0.0945	0.041*	
C2	0.9122 (6)	0.7015 (5)	−0.0844 (4)	0.0410 (9)	
H2A	0.9373	0.7376	−0.1521	0.049*	
C3	0.7906 (6)	0.5522 (4)	−0.1286 (4)	0.0395 (9)	
H3A	0.7343	0.4850	−0.2269	0.047*	
C4	0.6230 (5)	0.3430 (4)	−0.0625 (4)	0.0375 (9)	
H4A	0.5630	0.2713	−0.1593	0.045*	
C5	0.5901 (5)	0.2991 (4)	0.0411 (4)	0.0374 (9)	
H5A	0.5115	0.1968	0.0150	0.045*	
C6	0.6387 (5)	0.3701 (4)	0.3039 (4)	0.0363 (9)	
H6A	0.5605	0.2695	0.2834	0.044*	
C7	0.7191 (6)	0.4816 (4)	0.4423 (4)	0.0371 (9)	
H7A	0.6937	0.4580	0.5167	0.045*	
C8	0.8392 (5)	0.6306 (4)	0.4720 (4)	0.0324 (8)	
H8A	0.8933	0.7053	0.5673	0.039*	
C9	0.7490 (5)	0.4984 (4)	−0.0256 (3)	0.0289 (8)	
C10	0.6746 (5)	0.4079 (4)	0.1915 (4)	0.0285 (7)	
C11	0.8398 (5)	0.6068 (3)	0.1210 (3)	0.0235 (7)	
C12	0.7988 (5)	0.5611 (3)	0.2306 (3)	0.0230 (7)	

### Atomic displacement parameters (Å^2^)

**Table:**

	*U*^11^	*U*^22^	*U*^33^	*U*^12^	*U*^13^	*U*^23^
Co1	0.0244 (3)	0.0210 (2)	0.0226 (3)	0.00471 (18)	0.00743 (18)	0.00793 (19)
N1	0.0317 (16)	0.0289 (15)	0.0245 (15)	0.0127 (13)	0.0077 (13)	0.0103 (13)
N2	0.0352 (18)	0.0192 (14)	0.0265 (16)	0.0055 (13)	0.0027 (14)	0.0020 (13)
N3	0.089 (3)	0.039 (2)	0.038 (2)	0.021 (2)	0.0026 (19)	0.0190 (17)
N4	0.0317 (18)	0.0273 (16)	0.0434 (18)	0.0082 (14)	0.0105 (15)	0.0146 (14)
N5	0.0326 (18)	0.0191 (14)	0.0332 (17)	0.0100 (13)	0.0167 (15)	0.0145 (13)
N6	0.0289 (17)	0.0355 (17)	0.0396 (18)	0.0050 (14)	0.0083 (15)	0.0195 (15)
N7	0.0248 (15)	0.0268 (15)	0.0252 (14)	0.0098 (12)	0.0112 (12)	0.0124 (12)
N8	0.0276 (15)	0.0216 (14)	0.0210 (14)	0.0072 (12)	0.0060 (12)	0.0081 (12)
C1	0.035 (2)	0.042 (2)	0.033 (2)	0.0161 (17)	0.0165 (17)	0.0215 (18)
C2	0.044 (2)	0.063 (3)	0.033 (2)	0.029 (2)	0.0211 (18)	0.027 (2)
C3	0.038 (2)	0.049 (2)	0.0268 (19)	0.0207 (19)	0.0093 (17)	0.0091 (18)
C4	0.033 (2)	0.033 (2)	0.030 (2)	0.0162 (17)	0.0035 (17)	−0.0019 (17)
C5	0.032 (2)	0.0247 (18)	0.042 (2)	0.0091 (16)	0.0064 (17)	0.0047 (17)
C6	0.032 (2)	0.0249 (18)	0.049 (2)	0.0061 (16)	0.0136 (18)	0.0165 (18)
C7	0.044 (2)	0.035 (2)	0.040 (2)	0.0110 (17)	0.0211 (18)	0.0239 (18)
C8	0.035 (2)	0.0322 (19)	0.0281 (18)	0.0114 (16)	0.0095 (16)	0.0130 (16)
C9	0.0287 (19)	0.0333 (19)	0.0232 (17)	0.0181 (16)	0.0068 (15)	0.0075 (15)
C10	0.0244 (18)	0.0258 (17)	0.0332 (19)	0.0127 (14)	0.0080 (15)	0.0096 (15)
C11	0.0223 (17)	0.0235 (17)	0.0224 (17)	0.0092 (14)	0.0071 (14)	0.0078 (14)
C12	0.0179 (16)	0.0211 (16)	0.0265 (17)	0.0074 (13)	0.0059 (14)	0.0080 (14)

### Geometric parameters (Å, º)

**Table:**

Co1—N1^i^	2.113 (3)	C2—C3	1.356 (5)
Co1—N1	2.175 (3)	C2—H2A	0.9300
Co1—N4	2.202 (3)	C3—C9	1.416 (5)
Co1—N6^ii^	2.144 (3)	C3—H3A	0.9300
Co1—N7	2.141 (3)	C4—C5	1.347 (5)
Co1—N8	2.141 (3)	C4—C9	1.431 (5)
N1—N2	1.209 (4)	C4—H4A	0.9300
N1—Co1^i^	2.113 (3)	C5—C10	1.440 (5)
N2—N3	1.155 (4)	C5—H5A	0.9300
N4—N5	1.175 (4)	C6—C7	1.359 (5)
N5—N6	1.178 (4)	C6—C10	1.411 (5)
N6—Co1^ii^	2.144 (3)	C6—H6A	0.9300
N7—C1	1.329 (4)	C7—C8	1.386 (5)
N7—C11	1.364 (4)	C7—H7A	0.9300
N8—C8	1.339 (4)	C8—H8A	0.9300
N8—C12	1.362 (4)	C9—C11	1.408 (4)
C1—C2	1.411 (5)	C10—C12	1.403 (4)
C1—H1A	0.9300	C11—C12	1.434 (4)
			
N1^i^—Co1—N8	97.49 (11)	C3—C2—H2A	120.3
N1^i^—Co1—N6^ii^	93.69 (12)	C1—C2—H2A	120.3
N8—Co1—N6^ii^	167.62 (10)	C2—C3—C9	120.1 (3)
N1^i^—Co1—N7	168.99 (10)	C2—C3—H3A	119.9
N8—Co1—N7	77.67 (10)	C9—C3—H3A	119.9
N6^ii^—Co1—N7	92.20 (11)	C5—C4—C9	120.9 (3)
N1^i^—Co1—N1	79.52 (11)	C5—C4—H4A	119.5
N8—Co1—N1	95.55 (10)	C9—C4—H4A	119.5
N6^ii^—Co1—N1	91.67 (11)	C4—C5—C10	121.1 (3)
N7—Co1—N1	91.02 (10)	C4—C5—H5A	119.5
N1^i^—Co1—N4	94.21 (11)	C10—C5—H5A	119.5
N8—Co1—N4	84.91 (10)	C7—C6—C10	119.7 (3)
N6^ii^—Co1—N4	89.00 (11)	C7—C6—H6A	120.2
N7—Co1—N4	95.19 (11)	C10—C6—H6A	120.2
N1—Co1—N4	173.73 (10)	C6—C7—C8	119.7 (3)
N2—N1—Co1^i^	127.0 (2)	C6—C7—H7A	120.2
N2—N1—Co1	121.2 (2)	C8—C7—H7A	120.2
Co1^i^—N1—Co1	100.48 (11)	N8—C8—C7	123.0 (3)
N3—N2—N1	179.2 (4)	N8—C8—H8A	118.5
N5—N4—Co1	128.3 (2)	C7—C8—H8A	118.5
N4—N5—N6	177.8 (3)	C11—C9—C3	116.7 (3)
N5—N6—Co1^ii^	118.0 (2)	C11—C9—C4	119.5 (3)
C1—N7—C11	118.1 (3)	C3—C9—C4	123.9 (3)
C1—N7—Co1	128.3 (2)	C12—C10—C6	117.2 (3)
C11—N7—Co1	113.60 (19)	C12—C10—C5	119.1 (3)
C8—N8—C12	117.6 (3)	C6—C10—C5	123.7 (3)
C8—N8—Co1	128.5 (2)	N7—C11—C9	123.1 (3)
C12—N8—Co1	113.6 (2)	N7—C11—C12	117.3 (3)
N7—C1—C2	122.5 (3)	C9—C11—C12	119.6 (3)
N7—C1—H1A	118.8	N8—C12—C10	122.9 (3)
C2—C1—H1A	118.8	N8—C12—C11	117.3 (3)
C3—C2—C1	119.4 (3)	C10—C12—C11	119.8 (3)
			
N1^i^—Co1—N1—N2	145.8 (3)	C1—C2—C3—C9	1.4 (5)
N8—Co1—N1—N2	−117.6 (2)	C9—C4—C5—C10	2.1 (5)
N6^ii^—Co1—N1—N2	52.4 (3)	C10—C6—C7—C8	−1.3 (5)
N7—Co1—N1—N2	−39.9 (3)	C12—N8—C8—C7	0.4 (5)
N1^i^—Co1—N1—Co1^i^	0.0	Co1—N8—C8—C7	−172.4 (2)
N8—Co1—N1—Co1^i^	96.62 (12)	C6—C7—C8—N8	0.2 (5)
N6^ii^—Co1—N1—Co1^i^	−93.45 (12)	C2—C3—C9—C11	−0.1 (5)
N7—Co1—N1—Co1^i^	174.32 (11)	C2—C3—C9—C4	−179.9 (3)
N1^i^—Co1—N4—N5	−41.8 (3)	C5—C4—C9—C11	0.0 (5)
N8—Co1—N4—N5	−139.0 (3)	C5—C4—C9—C3	179.8 (3)
N6^ii^—Co1—N4—N5	51.8 (3)	C7—C6—C10—C12	1.7 (5)
N7—Co1—N4—N5	143.9 (3)	C7—C6—C10—C5	−177.8 (3)
N1^i^—Co1—N7—C1	111.0 (5)	C4—C5—C10—C12	−2.1 (5)
N8—Co1—N7—C1	175.8 (3)	C4—C5—C10—C6	177.5 (3)
N6^ii^—Co1—N7—C1	−11.4 (3)	C1—N7—C11—C9	1.7 (5)
N1—Co1—N7—C1	80.4 (3)	Co1—N7—C11—C9	−177.4 (2)
N4—Co1—N7—C1	−100.5 (3)	C1—N7—C11—C12	−177.9 (3)
N1^i^—Co1—N7—C11	−70.1 (6)	Co1—N7—C11—C12	3.0 (3)
N8—Co1—N7—C11	−5.2 (2)	C3—C9—C11—N7	−1.5 (5)
N6^ii^—Co1—N7—C11	167.6 (2)	C4—C9—C11—N7	178.3 (3)
N1—Co1—N7—C11	−100.7 (2)	C3—C9—C11—C12	178.1 (3)
N4—Co1—N7—C11	78.4 (2)	C4—C9—C11—C12	−2.1 (5)
N1^i^—Co1—N8—C8	−10.2 (3)	C8—N8—C12—C10	0.0 (4)
N6^ii^—Co1—N8—C8	144.2 (5)	Co1—N8—C12—C10	173.9 (2)
N7—Co1—N8—C8	179.9 (3)	C8—N8—C12—C11	178.6 (3)
N1—Co1—N8—C8	−90.3 (3)	Co1—N8—C12—C11	−7.6 (3)
N4—Co1—N8—C8	83.4 (3)	C6—C10—C12—N8	−1.1 (5)
N1^i^—Co1—N8—C12	176.8 (2)	C5—C10—C12—N8	178.5 (3)
N6^ii^—Co1—N8—C12	−28.8 (6)	C6—C10—C12—C11	−179.6 (3)
N7—Co1—N8—C12	6.8 (2)	C5—C10—C12—C11	0.0 (5)
N1—Co1—N8—C12	96.6 (2)	N7—C11—C12—N8	3.1 (4)
N4—Co1—N8—C12	−89.6 (2)	C9—C11—C12—N8	−176.5 (3)
C11—N7—C1—C2	−0.3 (5)	N7—C11—C12—C10	−178.3 (3)
Co1—N7—C1—C2	178.6 (2)	C9—C11—C12—C10	2.1 (5)
N7—C1—C2—C3	−1.2 (5)		

Symmetry codes: (i) −*x*+2, −*y*+2, −*z*+1; (ii) −*x*+3, −*y*+2, −*z*+1.
